# Supplementary material for: Food taboos and related misperceptions during pregnancy in Mekelle city, Tigray, Northern Ethiopia
Source: PLoS One. 2020 Oct 13;15(10):e0239451. doi: 10.1371/journal.pone.0239451 (PMC7553351; doi:10.1371/journal.pone.0239451)
Supplement: S1 File — (DOCX) [file pone.0239451.s001.docx]

# **Questionnaire for data collection**

## **English version**

### **Introduction and Consent Form**

Hi madam! My name is ________and I am here to collect data for the purpose of assessing food taboos and related misconceptions among pregnant women attending ANC from the clinics found in Mekelle city. The information you provide will us help identify foods that are wrongly prohibited during pregnancy for the improvement of the maternal and child health and nutrition. You are recruited to participate in the study just by chance. You will be interviewed on some questions for about 15 minutes. You have the right to participate in, refuse as well as drop out of the study at any time you want. Whatever information you provide will not be shared with anyone other than members of our study team. The research has no any associated risk and no compensation will be given for the time you spent with us. But you will be counseled regarding healthy eating during pregnancy and you have the right to know the findings of this study. I hope you will agree to participate since your information is important to the successful accomplishment of the study.

If you have questions regarding this study or would like to be informed of the results after its completion, please feel free to contact the principal investigator with phone number: - +251-919074746

May I begin the interview now? 1. Agree 2. Not agree

Proceed if respondent agrees, stop if she didn’t agree.

Thank you for your willingness to participate in this study.

| Address/Kebelle______________________Name of health facility______________________  Date of interview _____________Time started___________ Time finished_____________  Code number_________ | | | | | | | |  |  |  |
| --- | --- | --- | --- | --- | --- | --- | --- | --- | --- | --- |
| **Socio-demographic Characteristics** | | | | | | | |  |  |  |
| **Question** | **Response/ value** | | | | | **Remark** | | | |  |
| Age of the mother | -------------years | | | | |  | | | |  |
| Ethnicity? | 1.Tigray  2.Amara  3.Afar  4.Other(specify)______ | | | | |  | | | |  |
| Place of residence | 1. Urban 2. Rural | | | | |  | | | |  |
| Religion | 1.Orthodox  2.Muslim  3.Catholic  4.protestant  5.Others | | | | |  | | | |  |
| Current marital status | 1.Single  2.Married  3. Divorced  4. Widowed  5.Other(specify)______ | | | | |  | | | |  |
| Educational level? | 1.illitrate  2.primary education  3.secondary education  4.diploma  5.degree and above | | | | |  | | | |  |
| Current occupational status? | 1.Student  2.Government employee  3. private employee  4.NGOs employee  5.Self-employed  6.House wife  7.day laborer  8.Seeking for job  9.Other(specify)_____ | | | | |  | | | |  |
| Total family members | __________in number | | | | |  | | | |  |
| Who is decision maker to use money for living activities? | 1. mother  2. father  3.Both jointly  4.Others (specify)_____ | | | | |  | | | |  |
| How many time did you give birth? | | _______in number | | | |  | | | |  |
| What is the type the current pregnancy? | | 1. Unplanned and Unwanted  2. Unplanned but wanted  3. Planned and Wanted  4. I don’t know | | | |  | | | |  |
| **Nutrition and behavioral related factors** | | | | | | | | | |  |
| What are your staple foods? | | | 1. Teff injera and wet  2. Maize and Sorghum  3. Spagati and Rice  4. Vegetables/ fruits  5. Others__________ | | | |  | | | |
| Do you take additional food than usual? | | | 1. No  2. Yes | | | |  | | | |
| How many times do you eat per day when non-pregnant? | | | 1. Three times  2. Four times  3. more than four times | | | |  | | | |
| How many times do you eat per day at this time, when you are pregnant? | | | 1. Three times  2. Four times  3. more than four times | | | |  | | | |
| Are there any foods forbidden for pregnant mothers in your culture? | | | 1. No  2. Yes | | | | If no skip to Q. 505 | | | |
| If yes to #Q303 Please mention them_________________________ | | | | | | |  | | | |
| Why they are forbidden? __________________________________ | | | | | | |  | | | |
| Did you fast during your pregnancy? | | | | 1. No 2. Yes | | |  | | | |
| If the answer for no 506 is yes; What do you fast? | | | | 1. Animal products 2. Animal products and fast foods until 6 o’clock 3. Animal products and fast foods throughout the day | | |  | | | |
| What is the status of your appetite (eating condition) during pregnancy? | | | | 1. Decreased  2. Increased  3. No change | | |  | | | |
| Do you have any smoking history during the current pregnancy? | | | | 1. No 2. Yes | | |  | | | |
| Do you have any alcohol drinking history during the current pregnancy? | | | | 1. Yes 2. No | | |  | | | |
| Physical exercise per day | | | | 1. Not at all 2. 30 minutes or more 3. Less than 30 minutes | | |  | | | |
| **Questions on women dietary diversity (WDD)**  Did you eat any of the following foods (meal or snack) during the past 24 hours? | | | | | | | |  |  |  |
| Did you eat any bread, biscuits, or any other foods made from cereal (maize, sorghum, millet, wheat, barely or Teff) or white potatoes, white cassava, or other foods made from roots yesterday? | | | | | 1. Yes 2. No | | | |  |  |
| Did you eat any vegetable such as pumpkin, carrot, yellow/orange flesh sweet potatoes, or any fruit like ripe mango, papaya, banana, avocado, lemon, orange or 100% juice made from them yesterday? | | | | | 1. Yes 2. No | | | |  |  |
| Did you eat any dark green leafy vegetables (kale, cassava leaves, Swiss chard, cabbage) yesterday? | | | | | 1. Yes 2. No | | | |  |  |
| Did you eat other vegetables (e.g. tomato, onion, or other locally available vegetables or other fruits, including wild fruits and 100% fruit juice made from these yesterday? | | | | | 1. Yes 2. No | | | |  |  |
| Did you eat any meat (beef, lamb, and goat, chicken) or fish and other seafood yesterday? | | | | | 1. Yes 2. No | | | |  |  |
| Did you eat any eggs yesterday? | | | | | 1. Yes 2. No | | | |  |  |
| Did you eat any organ meat (liver, kidney, and heart) yesterday? | | | | | 1. Yes 2. No | | | |  |  |
| Did you eat any food made from legumes like kidney beans, haricot beans, field peas, cow peas, chick peas, nuts, lentils or others? | | | | | 1. Yes 2. No | | | |  |  |
| Did you eat any milk, cheese, yogurt and other milk products yesterday? | | | | | 1. Yes 2. No | | | |  |  |

## **Tigrigna (local language) version questionnaire**

**ናይ ምፍላጥን ስምምዕነትን ቅጽ**

ሰላም ዳሓንዶ ዊዒለን/ሓዲረን？ ሽመይ________________ ይበሃል። አብዚ ዘለኹሉ ምኽንያት አብ እዋን ጥንሲ ዝክልከሉ ምግብታትን ምክንያታቶምን ዝድህስስ ሓበሬታ ንምእካብ እዩ። ናብዚ መጽናዕቲ ዝተሓረኽሉ ምኽንያት ብዕጫ ወይ ድማ ብዕዱል እንትኸዉን እዚ እትህብና ሓበሬታ ድማ አብ ግዘ ጥንሲ ንዝህሉ ዘይተደለየ ምክልካል ዓይነት ምግቢ ብምስትክካል ጥዕና እታ ኣዶን ዕሸልን ምምሕያሽ እዩ። ፍቃደኛ እንተድአ ኮይንኪ አብዚ ዛዕባ አመልኪተ ካብ 15 ደቃይቅ ንዘይበዝሕ ግዘ ቃለ መሕተት ክገብረልኪ እየ። አብዚ ጽንዓት እዚ ናይ ምስታፍን ዘይምስታፍን ኮነ አብ ማእኸል ናይ ምቁራጽ መሰልኪ ዝተሓለወ እዩ። እትህብኒ ሓበሬታ ካብ ናይዚ ጽንዓት ጉጅለ ወጻኢ ካልኦት ወገናት ንኸይፈልጥዎ ምስጢሩ ዝተሓለወ እዩ። እዚ ጽንዓት እዚ አባኺ ዋላ ሓደ ዘብጽሖ ጉድአት የለን፤ ምሳይ ንእተሕልፍዮ ግዜ እዉን ዝኽፈለኪ ምንም ዓይነት መክሓሓሲ የለን። አብ ግዜ ጥንሲ ክህሉ ስለዘለዎ ስሩዕ ኣመጋግባ ምክርን ማዕዳን ክዋሃበኪ እዩ። ዉጽኢት ናይዚ መጽናዕቲ እዉን ናይ ምፍላጥ መሰልኪ ሕልዉ እዩ። እትህብና ሃበሬታ ነዚ መጽናዕቲ ብጣዕሚ ጠቃሚ ስለዝኮነ ንምስታፍ ፍቃደኛ ከምትኾኒ በዓል/ቲ ሙሉእ ተስፋ እየ። ስለዚ ጽንዓት ዝምልከት ግልጺ ክኾነልኪ እትደልዪዮ ነገር እንተሃልዩ ንናይዚ ጽንዓት በዓልቲ ዋና በዚ ዝስዕብ አድራሻ ደዊልኪ ምሕታት ትኽእሊ ኢኺ: +251919074746

ቃለ መሕተት ክጅምር ይኽእል ድየ？ 1. እሺ 2. አወይፋሉን

ተሳታፊት እንተዳኣ ተስማዕሚዓ ቃለ መሕተት ጀምር።

ንምስታፍ ፍቃደኛ ስለዝኾንኪ አዝየ የመስግን።

| ናይ መሕተት መለለይ ቁጽሪ_____________ አድራሻ_____________ሽም ጥዕና ትካል_____________  ዕለት___________ዝተጀመረሉ ሰዓት___________ ዝተወደአሉ ሰዓት__________ኮድ ቁጽሪ_______________ | | |
| --- | --- | --- |
| **ክፋል 1：ማሕበራዉን ኢኮነምያዉን ኩነታት** | | |
| ሕቶ | መልሲ | መብርሂ |
| ናይ አዶ ዕድመ | __________ብዓመት |  |
| ብሄር | 1. ትግራይ  2. አምሓራ  3. ዓፋር  4. ካልእ（ጸሓፍ ——— |  |
| እትነብርሉ ቦታ | 1. ከተማ  2. ገጠር |  |
| ሃይማኖት | 1. ኦርቶዶክስ  2. ሙስሊም  3. ካቶሊክ  4. ፕሮቲስታንት  5. ካልእ———— |  |
| ኩነታት መርዓ | 1. ዘይተመርዐወት   1. ዝተመርዐወት   3. ዝተፋትሐት  4. ሰብአያ ዝሞታ  5. ካልእ———— |  |
| ደረጃ ትምህርቲ | 1. ዘይተምሃረት  2. ቀዳማይ ደረጃ  3. ካልአይ ደረጃ  4. ዲፕሎማ  5. ዲግሪ ና ከዛ በላይ |  |
| ኩነታት ስራሕ | 1. ተምሃሪት  2. ሰራሕተኛ መንግስቲ  3. ሰራሕተኛ ዘይመንግስታዊ ትካል  4. ዓርሰ ቁጻር  5. ናይ ገዛ እመቤት  6. መዓልታዊ ሰራሕተኛ  7. ካልእ_________ |  |
| ወርሓዊ ናይ ስድራ እቶት | ——————ቅርሺ |  |
| በዝሒ ስድራ | —————— ብቁጽሪ |  |
| ናይ ገዛ ሰራሕተኛ/ሞግዚት አላትኪ ዶ？ | 1. አይፋሉን  2. እወ |  |
| ቅርሺ ዝዉንን መራሒ ስድራ መን እዩ ？ | 1. አቦ 2. አዶ 3. ክልቲኦም |  |
| ክሳብ ሕዚ ክንደይ ቆልኡ ወሊድኪ？ | —————— ብቁጽሪ |  |
| ናይ ሕዚ ጥንስኪ እንታይ ዓይነት እዩ？ | 1. ዘይተደለየን ዘይተተለመን  2. ዝተደለየ ግን ድማ ዘይተተለመ  3. ዝተተለመን ዝተደለየን  4. አይፈልጦን |  |
|  | | |

**ክፋል 2: ናይ አመጋግባን ባህርይን ኩነታት/ Nutritional behavioral factors**

| አዘዉቲርኪ እትምገብዮ ዓይነት ምግቢ እንታይ እዩ？ | 1 እንጀራ ጣፍ  2 ካብ ምሸላን ምሻሓርን ዝተሰርሐ እንጀራ  3 ፓስታን ሩዝን  4 አትክልትን ፍራምረን  5 ካልእ————— | |  |
| --- | --- | --- | --- |
| ቅድሚ ምጥናስኪ ብመዓልቲ ክንደይ ግዜ ትምገቢ ኔርኪ | 1 ሰለስተ  2 አርባዕተ  3 ልዕሊ አርባዕተ | |  |
| አብ ናይ ሕዚ ግዜ ጥንስኺ ብመዓልቲ ክንደይ ግዜ ትምገቢ? | 1 ሰለስተ  2 አርባዕተ  3 ልዕሊ አርባዕተ | |  |
| ብባህልኹም ንጥኑሳት አዴታት ዘይዉሃብ ዓይነት ምግቢ አሎዶ？ | 1 አይፋሉን  2 እወ | | አይፋሉን፤  ናብ ቁ 508 ስገር |
| ንሕቶ ቁጽሪ 503 መልሱ እወ እንተኾይኑ፤ እቶም ዘይወሃቡ ዓይነታት ምግቢ ዘርዝር———————— | | | |
| ንምንታይ ከ እዮም ዘይወሃቡ?____________________________________________________ | | | |
| አብ ግዜ ጥንስኺ ትጾሚ ዶ？ | | 1 አይፋሉን  2 እወ | አይፋሉን፤  ናብ ቁ 510 ስገር |
| ንሕቶ ቁ 506 እወ እንተኾይኑ እንታይ እንታይ ኢኺ ትጾሚ？ | | 1 ናይ እንስሳት ተዋጽኦ ጥራሕ  2 ናይ እንስሳት ተዋጽኦን ናይ ጾም ምግቢ ክሳብ ሽድሽተ ሰዓትን  3 ካልእ—————— |  |
| አብ ግዜ ጥንሲ（ትማሊ ዕለት） ናይ ምግቢ ድልየትኪ ከመይ እዩ？ | | 1 ይቅንስ  2 ይዉስኽ  3 ለዉጢ የብሉን |  |
| አብናይ ሕዚ ጥንስኺ ግዜ ሽጋራ አትኪኽኪ ትፈልጢ ዶ？ | | 1 አይፋሉን  2 እወ |  |
| አብናይ ሕዚ ጥንስኺ አልኮላዊ መስተ ሰቲኺ ትፈልጢ ዶ？ | | 1 አይፋሉን  2 እወ |  |
| ብመዓልቲ ክንደይ ዝአክል እንቅስቃሴ ትገብሪ？ | | 1 ምንም አይንቀሳቀስን  2 ትሕቲ 30 ደቂቃ  3 ልዕሊ 30 ደቂቃ |  |

**ክፋል 3: ብጥንስቲ አዶ ዝብልዑ ዒይነታት ምግቢ ዝድህስስ መጠይቅ：ኣብ ዝሓለፉ 24 ሰዓታት ውሽጢ ካብዞም ዝስዕቡ ዓይነታት ምግቢ ኣየነኦም ተመጊብክን？**

| ሕቶ | መልሲ |
| --- | --- |
| ትማሊገሊኦም ካብ ጥረምረ ማለት እዉን ካብ ዒልቦ፣ ምሸላ፣ ስገም፣ ስርናይ፣ጣፍ ዝተሰርሐ ቅጫ፣ እንጀራ፣ ጋዓት፣ ሕምባሻ፣ ብሽኩቲ ዝኾነ ዓይነት ምግቢ ወይ ክም ድንሽን ካልኦት ሱራምርን ዝሓዘ ምግቢ ተመጊብኪ ዶ ኔርኪ？ | 1 አይፋሉን  2 እወ |
| ትማሊ ገሊኦም ከም ድባ፣ ካሮት፣ ዝኾነ ዒይነት ሽኮር ድንሽ፣ ክቢ ድንሽ፣ ሽጉርቲ ወይ ከዓ ካልእ ሱራምር ዝሓዘ ምግቢ ወይ ድማ ገሊኦም ፍራምረ ከም ዝበሰለ ማንጎ፣ ፓፓየ፣ ሙዝ፣ ኣቮካዶ፣ ሎሚን፣ ኣራንሺ ወይ 100% ካብዚኦም ዝተሰርሐ ጽሟቅ ተመጊብኪ ዶ ኔርኪ？ | 1 አይፋሉን  2 እወ |
| ትማሊ ገሊኦም ካብ ሓምለዎት ቆፅሊ መፅሊ ከም ሓምሊ-አድሪ፣ ቁስጣ፣ ስላጣ፤ ወይ ካውሎ ዝሓዘ ምግቢ ተመጊብኪ ዶ ኔርኪ？ | 1 አይፋሉን  2 እወ |
| ትማሊ ገሊኦም ካብ ካልኦት አሕምልቲ ከም ኮሚደረ፣ ሽጉርቲን ካሌኦትን ዝሓዘ ምግቢ ወይ ከዓ ገሊኦም ካብ ናይ በረኻ ፍራምረ ወይ ካብአቶም ዝተሰርሐ 100% ጽሟቅ ተመጊብኪ ዶ ኔርኪ？ | 1 አይፋሉን  2 እወ |
| ትማሊ ገሊኦም ካብ ውፅኢት ስጋ ከም ናይ ከፍቲ፣ ጤል፣ በጊዕን ደርሆን ወ ይ እዉን ዓሳ በሊዕኺ ዶ ኔርኪ？ | 1 አይፋሉን  2 እወ |
| ትማሊ ከም ናይ እንስሳ ፀላም ከብዲ፣ ኩሊት ወይ ልቢ በሊዕኺ ዶ ኔርኪ？ | 1 አይፋሉን  2 እወ |
| ትማሊ እንቋቁሖ በሊዕኺ ዶ ኔርኪ？ | 1 አይፋሉን  2 እወ |
| ትማሊ ገሊኦም ካብ ዓይነታት ዓተር ከም ዓልቋይ፣ ዓይኒዓተር፣ ባሎንጋ፣ ሽምብራ፣ብርስን ካልኦትን ዝተሰርሐ ምግቢ በሊዕኺ ዶ ኔርኪ？ | 1 አይፋሉን  2 እወ |
| ትማሊ ገሊኦም ካብ ፀባን ውፅኢት ፀባ ከም ርግኦን ኣጅቦን ዝበሉ በሊዕኺ ዶ ኔርኪ？ | 1 አይፋሉን  2 እወ |
