# Supplementary material for: Food taboos and related misperceptions during pregnancy in Mekelle city, Tigray, Northern Ethiopia
Source: PLoS One. 2020 Oct 13;15(10):e0239451. doi: 10.1371/journal.pone.0239451 (PMC7553351; doi:10.1371/journal.pone.0239451)
Supplement: S2 File — (DOCX) [file pone.0239451.s002.docx]

## **Sampling technique**

Five private clinics namely, Kalkidan hospital, Ruwet, Bethlehem, Ado and Se-A-Me clinics were selected based on their case flow. Using the flow of pregnant women who visit ANC services in the selected clinics in the same three months of the previous year as a baseline, samples were proportionally allocated to each clinic. Using this information as baseline data, the total number of clients that were included in the study from each clinic was calculated as follows:

**Average number of mothers visiting ANC services in a month from a specific clinic × sample size**

**Total antenatal attendance for the five health facilities over the same month**

All mothers coming to these clinics during the study period were taken consecutively until the total sample size is attained.

Flow chart that shows the proportional allocation of pregnant women to each of the selected clinics
