# Supplementary material for: Food taboos and related misperceptions during pregnancy in Mekelle city, Tigray, Northern Ethiopia
Source: PLoS One. 2020 Oct 13;15(10):e0239451. doi: 10.1371/journal.pone.0239451 (PMC7553351; doi:10.1371/journal.pone.0239451)
Supplement: Attachment — Submitted filename: PONE-D-20-06088_VS_SFA_23 March 2020.docx [file pone.0239451.s004.docx]

Remarks to the Manuscript by **Tela FG** et al. on

**„Food taboos and related misperceptions during pregnancy in Mekelle city, Tigray, Northern Ethiopia“**

We strongly agree that the level and impacts of food taboos on the health of pregnant mothers, fetus and pregnancy outcome need to be explored. To reduce its related negative impacts, sound and appropriate evidence informed interventions need to be implemented. In this sense, we appreciate the researchers for investigating and bringing scientific information on food taboos and related misconceptions among pregnant mothers of Mekelle city.

**Abstract:**

Ln 27: Add “in” after the word “limited” and before the word “Ethiopia”.

Ln 30-31: the timeline should be specified as “1^st^ of January to 30^th^ of June 2017”.

- However, we wonder why this timeline of data collection is different from what is mentioned on Ln 87, where it was stated that “*The study was carried out from April to May 2017*….”. Which one is correct?

Ln 33/34: please, do the following changes: “described” to “presented”, “frequency” to “frequencies” and “percentage to “percentages”. Check also Ln144-145.

Ln 43: you may add “Ethiopia” as a keyword.

**Introduction**

Ln 55-59: The sentence is very long and it needs to be broken-down in two short and informative sentences.

Ln 56: “under-nutrition” should be written without hyphen as “undernutrition”

Ln 60-63: please, cite the references because when you say “…have been reported…”, you are referring to previous research facts, not of yours.

Ln 67: add comma (,) after the word, “especially”, and why is it “especial”? it could be a good idea if you can paraphrase this sentence.

Ln 76: the word “closely” is a very ambiguous expression and you may need to replace it by “negatively” or any other clear word, which can clearly indicate the nature &/ or direction of the association.

Ln 79: avoid the semicolon (;) and better replace it by comma (,).

**Methods**

Ln 83: The “Study period” sub-section could be mentioned together with the “Study period” under one sub-topic, written as “Study design and period”. Then, the section “Study design and period” should come after the “Study area”.

- Under the “Study design and period”, the study objective must not be mentioned here. Rather, you may paraphrase as in the following: “A descriptive cross-sectional study was conducted from April to May 2017 (make sure to put the correct timeline).”

Ln 87: The study period referred here is different from the one mentioned in Ln 30-31. Timing is a very important dimension in epidemiologic studies. Why is it different? Which one is correct?

Ln 89-92: What is the source of the information? Please, cite the source. What does “2016/2017” mean? Does it refer for either of the years or for both?

Ln 91-92: The sentence is unclear and it may mean that all these health stations are there only to provide ANC services. Please, paraphrase it.

Ln 101: for your future research plan, you need to know three important points regarding sample size calculation. First, if your calculated sample size has a decimal, the sample size must be ROUNDED UP regardless of the value of the decimal. Many researchers do not do this, but from a statistical point of view; this is what has to be done. For instance, in your case, the calculated sample size it **332.2** and it has to be approximated up and it should be **333**. Second, whenever you are considering “none response rate”, you should calculate it using the formula (sample size divided by the response rate in decimal) and it should not be added just by taking 10% of the calculated sample size. In your case, the initial actual sample size is 302.9 (≈303) and taking a 10% non-response rate (303/0.90= 33.7≈ 34, and the final sample size (at its best scenario) would have been 337. Third, if your source population (in this case the total number of pregnant women in the study area) is small (<10,000), the sample size need to take in to account, a population correction factor. Based on your report for the year prior to your study, your study population is not a small population (check Ln 91).

Ln 97: You may replace “Eligibility criteria” by “Inclusion and exclusion criteria”.

Ln 107: The sub-topic “sampling techniques” could be modified as “Sampling techniques and procedures”. What sampling technique was used? It is always important to mention the sampling method. From the texts, it seems that you have employed a stratified random sampling technique. You may address the following major concerns.

- Ln 108: Because your study clinics were randomly selected from the institutions with a “*better flow* of pregnant women for ANC services”, what does this imply to your findings? There could be the possibility of “selection bias” because participants of your study were from clinics, which had a “*better flow*” of pregnant women for ANC services. The profile (socio-demographic and economic attributes) of the participants of your study may differ from those who attended other health institutions, which did not have a “better flow”, and the prevalence of food taboo and related misconceptions could be different. You may discuss it.
- Ln 108: it may be better to say “ANC clinics with a higher attendance rate”.
- Ln 109 and Ln 112: If the allocation to each of the five health institutions was proportional. This implies the total number of pregnant women was known for each of these study clinics. How was the proportional allocation made? How was the sampling interval defined (N/n)? For a clarity purpose, you may submit a “supplementary” table or flowchart that shows the proportional allocation and its respective sampling interval.

Ln 120: you may modify the sub-topic to “Data collection tools and procedures”.

- Ln 122: “ in the way that they can address” may be replaced by “to asses “. You should replace “literatures” by “literature”.
- Ln 125: Your results show no behavioral factors. So, you may make it clear.
- Ln 132: contractions, like “didn’t”, should be avoided in scientific writing. Replace “didn’t” by “did not”.

Ln 135: Please, mention the number of data collectors and supervisors.

Ln 136: You may replace “overall purpose and methodology” by “overall purpose and data collection procedure of the study” if this makes sense to you.

Ln 137: On how many women and where was the pilot test implemented? Who were the actual participants of the pilot-test study?

Ln 137-139: It is great that you did the translations and retranslations. Who did both translations to the local language and the retranslation back to English? Was it done by the same individual/s? You may briefly explain these points.

Ln 145: add comma after “Finally”.

Ln 145: replace “cross-tabulations” by “Chi-square”. As shown in your table (check Ln 202), you have zero cell counts. In this case, you should use Fishers Exact Test as an alternative to chi square test. You should write the statistical parameter you used to summarize for the continuous variables.

- We strongly suggest using appropriate statistical analysis to identify the factors associated with the food taboo, which has a prevalence of 11.5%. Authors should use the data to its maximum potential and may have ethical aspect from a statistical point of view. We suggest to the authors to address two important points (based on their result from Table 4). First, they should combine the levels categories of some independent in to broader categories to avoid the problem of convergence. Second, they should test for presence of multicollinearity, which could bias their measure of association like the odds ratio or prevalence ratio. This is not a must to do, but we encourage you to do it.
- If the authors decide to run a model to identify the factors associated with food taboo, the objective and methods part of the study need to be modified accordingly.

**Results**

- **General comment: use present tense when referring to tables, figures and graphs. Otherwise, you must use simple past tense to report your results.**
- **Avoid using the word “significant”. Nowadays, it is highly encouraged using other terms like “increased or decreased” if your finding is statistically significant.**

Ln 160: Age of participants was reported in mean and standard deviation. Was it normally distributed? If not, you need to report the median and interquartile range. This works for all continuous variables you have (like family size).

Ln 162: replace “were diploma and above” by “had diploma and above”.

Ln 169-171: Check subject-tense agreement (simple past tense should be used). Replace “eat” to “ate”, and “get” by “got”.

Ln 185: avoid the comma (,) after the word “beans”

Ln 191: avoid “also” which is mentioned after “Honey was” because you have used it in the next sentence.

Ln 198-199: replace “are” by “were”.

Ln 180: report the 95% confidence interval of the prevalence.

Ln 198-199: replace “are” by “were”.

Ln 202: please, make sure that you reported Fishers Exact Test for the cell counts with zero values or the expected value is less than 5.

**Discussion**

- Generally, your findings are well discussed, but if you need to run additional statistical analysis, the discussion section will certainly need additional interpretations based on the new outputs.

Ln 249-253. This lacks critical interpretation of the results to create awareness on the potential harms of consuming honey on the health of their newborns. You should also critically discuss the various negative aspects of honey use. You need to cite more relevant articles in this field, which can help you to improve the respective discussion.

- Please, cite more important articles, you should at least cite the work of Ajibola et al. (doi: 10.1186/1743-7075-9-61)
- Natural honey can be contaminated by *c.botulinum,* which is fatal, and definitely, it should not be given to children less than 1 year. Check CDC’s official webpage (<https://www.cdc.gov/botulism/>)
