# Supplementary material for: Food taboos and related misperceptions during pregnancy in Mekelle city, Tigray, Northern Ethiopia
Source: PLoS One. 2020 Oct 13;15(10):e0239451. doi: 10.1371/journal.pone.0239451 (PMC7553351; doi:10.1371/journal.pone.0239451)
Supplement: Attachment — Submitted filename: Response to reviewers.docx [file pone.0239451.s005.docx]

**Response to reviewers**

**Comments raised by the academic editor:**

1. Please ensure that your manuscript meets PLOS ONE' style requirements, including those for file naming.

**Response: -** Thank you so much for your credible concern dear! I have read the PLOS ONE' guideline and revised the manuscript as much as possible.

2. Please include additional information regarding the survey or questionnaire used in the study and insure that you have provided sufficient details that others could replicate the analysis. For instance, please include a copy of the questionnaire also in the original language as supporting information. Moreover, please include more details on how the questionnaire was originated and pre-tested, and whether it was validated.

**Response: -** Thank you for your valid comment dear! I had wrongly attached inappropriate draft of the questionnaire; but now I have attached the right one in its English and Tigrigna (the local language) version as a supporting information. The questionnaire was developed by reviewing different literature. To be honest, the questionnaire was not validated; as there were some studies conducted in Ethiopia that used similar data collection tool, we simply reviewed these literature and developed the questionnaire.

3. We not that you have indicated that data from this study are available up on request. Plose only allows data to be available up on request if there are legal or ethical restrictions on sharing data publicly. In your revised cover letter, please address the following prompts:
   a. If there are ethical or legal restrictions on sharing a de-identified data set, please explain them in detail (example: data contain potentially identifying or sensitive patient information) and who has imposed them (an ethics committee). Please also provide contact information for a data access committee, ethics committee or other institutional body to which data requests may be sent.
   b. If there are no restrictions, please upload the minimal anonymized data set necessary to replicate your study finding as either supporting information files or to a stable, public repository and provide us with the relevant URLs, DOIs, or accession numbers.

**Response**: Sorry for the misinformation I made on behalf of all the authors dear! It was wrongly stated that data are available up on request. The data in this study have no ethical or legal restrictions on sharing publicly, and all data supporting the results of this study are made available in the manuscript and as supporting information. If I have forgotten anything important, I can provide it. I have incorporated this idea in the revised cover letter.

4. Thank you stating the following financial disclosure ‘’no funding’’
   a. Please provide an amended funding statement that declares *all* the funding or sources of support received during this specific study (whether external or internal to your organization) as detailed online for authors at <http://journals.plos.org/plosone/s/submit-now>

**Response**: Thank you for noticing this important issue dear! There is no any funding we received for this work. Furthermore, as the Plose guideline do not allow to incorporate funding statement in the manuscript, I have removed it up on revision of the manuscript.

b. Please state what role the funders took in the study. If any authors received a salary from any of your funders. Please state which authors and which funder. If the funders had no role, please state: ‘’the funders had no role in study design, data collection and analysis, decision to publish, or preparation of the manuscript.’’
- Please include your amended statements with in your cover letter; we will change the online submission form on your behalf.

**Response**: Thank you for your constructive comment dear! I will like to clarify that there were no funding in the overall accomplishment of this study including the study design, data collection and analysis, decision to publish, or preparation of the manuscript. We, the authors, made all the necessary efforts from the start to the final manuscript write-up, and no author received a salary from any funder. I have incorporated this idea in the revised cover letter.

**Reviewers’ comments:**

**Comments raised by reviewer 1:**

Thank you dear for your encouraging words and your relentless effort to boost the quality of our manuscript. All of your comments and suggestions are really appreciated and accepted!

1. The authors do almost nothing to situate the data reported with in a larger academic debate, and do not outline any theoretical perspective, specific hypothesis, or predictions. The most obvious perspective to bring to this manuscript is the developmental origins of health and disease (DOHaD) framework (which is sort of but not satisfactorily alluded to), although bio-cultural theory, dual inheritance theory, human behavioral ecology, evolutionary psychology, or some kind of network theory could also be compelling. Alternatively, an in depth ethnographic pharming describing the historical particulars (that is, the social as well as dietary significance of the food staff in the city and its sub-cultures) underpinning each of the common taboos would be interesting. Some intellectual framework needs to be established and justified for this to be publishable.

**Response**: Thank you very much for your valuable comments and suggestions dear. We have tried to refer articles that could explain the origin and background of food taboos and different theories associated with it as per your suggestion. Based on the scientific evidences that we have searched, we incorporated important points in the manuscript (lines 60 – 78)

2. The authors seemingly do not disentangle pregnancy food taboos from other kinds of food avoidances (visceral aversions, nausea, disgust, taboos not specific to pregnancy; avoidances due to public health guidelines; avoidances due to allergies or sensitivities or family preferences). It is not clear whether data were collected on whether pregnant people would usually eat a certain food and even wanted to eat it but did not because of a taboo or did not because it made them nauseous and there also happened to be a taboo about it. This issue needs to be better outlined in the methods; if data are available on other reasons for food avoidances, they should be reported and discussed; if data are not available, this should be discussed as a limitation and avenue for future research.

**Response**: Thank you for your invaluable and constructive comments dear! I will like to make it clear that we had clearly differentiated these terms in the very beginning of the study, and planned to assess the foods that are intentionally prohibited (taboo foods) during pregnancy for non-scientific reasons by the community in the study area. We have not collected a data on the other forms of food avoidances because we as public health nutrition professionals thought that we can easily make a public health intervention to address food taboos than the other forms of food avoidances. As per your suggestion, we have incorporated it in the limitation of the study (lines 317 – 325).

3. No alternatives to the assumption that the taboos are problematic were presented. Yes, the pregnant people are missing out nutrients, but are they also avoiding pathogens, or preventing fetal overgrowth, or guarantying that nutrients go to support other household members? I would expect to see some thoughtful discussion and familiarity with the literature around these points.

**Response**: We would like to appreciate for this valuable and important comment. It is true that food taboos would have positive and negative implications. As you mentioned it in your comment, people could avoid food related toxicity or poison through avoiding some food types. On the other hand, as we tried to focus in this study, food taboos would also have negative implications due to the risk of under nutrition. Therefore, we tried to address this issue that our main aim was to explore food taboos during pregnancy which could have negative implications (lines 79 – 85).

4. The authors do not seem to look at the extent to which pregnant people are perhaps compensating for nutrients through experiencing cravings or following other dietary recommendations and thus filling in some of the nutrient gaps created by food avoidances.

- My colleagues and I found that there was evidence of pregnant people in Figi eating other foods with similar nutrient profiles to avoid foods (Mckerracher et al. 2016; see also Henrich and Henrich 2010).

**Response**: Thank you for raising a very crucial point dear! We tried to address the foods believed by the community to be bad during pregnancy for non-scientific reasons which can compromise the daily dietary intake of the mothers eventually affecting the health and nutritional status of the mothers and their growing baby. As you can see from table 2 of the manuscript, more than 3/4^th^ (79.2%) of the pregnant women did not achieve the minimum dietary diversity score.

5. Education is a major predictor of not adhering to food taboos, and the public health recommendation the authors make then is to improve antenatal education (example lines 271 – 272). I would suggest going to pre-conception and even to adolescence to really make a big difference. See the work by MacNab and Mukisa in South Africa, by Jackie Bay and colleagues in Newzealand and the Cooc Islands, by Mary Barker/Kathy Townsend and colleagues with the Each-B trial in Southampton UK and so on. Also check out the Lancet review on pre-conception interventions (Stephenson et al. 2018).

**Response**: We have made some modification based on your suggestion dear. We thought that short and long term interventions are required to address the issue (lines 326 – 332).

6. I would generally like to see a little more context on the overall health of the pregnant population in Mekelle in general and in the sample in particular. What are infant and maternal mortality like? Are there high rates of GDM? High rates of pre-eclampsia/ Hypertension? High rates of fetal growth restriction? Pre-term birth? Average completed family size? Food security?

**Response**: Thank you very much for your valuable comment. We tried to search published scientific studies on these issues in the study area. However, we could not find studies regarding the magnitude of infant and maternal mortality rate, gestational diabetes mellitus, pre-eclamplsia/eclapsia, food security label, and fetal growth restrictions. We believe that these would be good areas of research that remained undiscovered. The average family size of the sample population were also 3.6 ≈ 4.

7. Please unpack the dietary diversity score. There should be clearly outlined in the main text.

**Response**: Thank you for raising a valid point dear! We have narrated the women diet diversity individually as per your suggestion (lines 210 – 216).

8. Lastly, the manuscript would benefit from a bit more spell and grammar checking. Hopefully, the editors can provide this or at least some financial assistance as the team is from a lower income country. I would also be happy to help with proofing the revision if I was sent a word doc. or Google doc. File.

**Response**: We tried to check the spelling and grammar of the whole manuscript as per your recommendation, and we would like to appreciate your willingness to help us in proofreading our manuscript and suggesting the editors to support us.

**Comments raised by reviewer 2:**

Thank you dear for your effort to make our manuscript better looking at it strictly and provision of constructive and invaluable comments. We accepted all of the comments as it is and here are the issues that need our response.

1. Line 30 – 31: the timeline should be specified as ‘’1^st^ of January to 30^th^ of June 2017. However, we wonder why this timeline of data collection is different from what is mentioned on line 87, where it was stated that ‘’the study was carried out from April to May 2017…’’ which one is correct?

**Response:** Sorry for the mistake we made in writing the timeline dear! But the one in the abstract which says from 1^st^ of January to 30^th^ of June indicates the timeline starting from proposal development up to the final accomplishment of the study. Whereas, the timeline in line 87 shows the period of data collection. Up on your suggestion, I have revised it and write the one which indicates the data collection period from April to May 2017 in both cases.

2. Line 89 – 92: what is the source of information? Please, cite the source. What does ‘’2016/2017’’ mean? Does it refer for either of the years or for both?

**Response**: Thank you for raising a very important point dear! We brought the information from the Federal democratic republic of Ethiopian central statistics agency report 2017. I have put a reference in line 108.

3. Line 108: because your study clinics were randomly selected from the institutions with a ‘’better flow of pregnant women for ANC services’’, what does this imply to your findings? There could be the possibility of ‘’selection bias’’ because participants of your study were from clinics, which had a ‘’better flow’’ of pregnant women for ANC services. The profile (socio-demographic and economic attributes) of the participants of your study may differ from those who attended other health institutions which did not have a ‘’better flow’’, and the prevalence of food taboo and related misconceptions could be different. You may discuss it.

**Response**: Thank you for your nice question dear! In the beginning (proposal write-up) of the study, we made an assessment of the clinics for the services they were providing, and the flow of the pregnant women being served. The services delivered by the different clinics (with better and relatively poor flow of pregnant mothers) were similar in terms of their quality and cost. Therefore, we thought that the difference in the flow of the mothers might be emanated from popularity of the health providers working in the clinics and peer pressure (mothers may go to the clinics where their friends or relatives are being served).

4. Line 109 and 112: if the allocation to each of the five health institutions was proportional, this implies the total number of pregnant women was known for each of these study clinics. How was the proportional allocation made? How was the sampling interval defined (N/n)? For a clarity purpose, you may submit a ‘’supplementary’ ’table or flow chart that shows the proportional allocation and its respective sampling interval.

**Response**: Thank you for raising a very important point. Now I have attached the clarification as a supplementary material up-on your suggestion.

5. Line 137: on how many women and where was the pilot test implemented? Who were the actual participants of the pilot test study?

**Response**: We conducted a pre-test rather than a pilot test dear. The questionnaire was pre-tested before the actual data collection on 5% (17) of the study participants in other similar clinics that were not included in the study. Some modifications were made based on the pretest results especially on the clarity of the questionnaire to the data collectors and respondents (lines 158 – 161).

6. Line 137 – 139: it is great that you did the translations and re-translations. Who did both translations to the local language and the re-translation back to English? Was it done by the same individual/s? You may briefly explain these points.

**Response**: Thank you for the noticing this nice point dear! The questionnaire was translated into Tigrigna (local language), and then back-translated to English by two individuals independently for ensuring the consistency of concepts (line 163).

7. We strongly suggest using appropriate statistical analysis to identify the factors associated with food taboo, which has a prevalence of 11.5%. Authors should use the data to its maximum potential and may have ethical aspect from statistical point of view. We suggest to the authors two important points (based on their result from table 4).

- 1^st^, they should combine the levels categories of some independent variables in to broader categories to avoid the problem of convergence.
- 2^nd^, they should test for presence of multi-collinearity, which could bias their measure of association like the odds ratio or prevalence ratio. This is not a must to do, but we encourage you to do it.
- If the authors decide to run a model to identify the factors associated with food taboo, the objective and methods part of the study need to be modified accordingly.

**Response**: A very nice suggestion dear! In the very beginning, our difficulty was to merge the variables. Now, we have run a new analysis by merging some of the variable in to broader categories up on your suggestion. We assessed the socio-demographic factors associated with food taboo practice among pregnant women using bivariate and multivariable logistic regression analysis. Accordingly, we made necessary modifications in the objective (lines 22 &23), methods (lines 168 – 185), result (lines 238 – 246), and discussion (lines 309 – 316) of the study.

8. Line 160: age of participants was reported in mean and standard deviation. Was it normally distributed? If not, you need to report the median and inter-quartile range. This works for all continuous variables you have (like family size).

**Response**: We have checked all of the continuous variables for normality using Shapiro Wilk’s test and they were found to be appropriately normally distributed (p> 0.05) (lines 170 – 173).

9. Line 180: report the 95% CI of the prevalence.

**Response**: Oh! Sorry for missing this important point dear! Thank you for seriously looking at everything in our manuscript dear! Now I have incorporated it (line 253)

10. Line 249 – 253: this lacks critical interpretation of the results to create awareness on the potential harms of consuming honey on the health of their newborns. You should also critically discuss the various negative aspects of honey use. You need to site more relevant articles in this field, which can help you to improve the respective discussion.
    a. Please site more important articles, you should at least site the work of Ajibola et al. (doi: 10.1186/1743-7075-9-61).
    b. Natural honey can be contaminated by C.botulinum, which is fatal and definitely, should not be given to children less than one year. Check CDC’s official webpage (<https://www.cdc.gov/botulism/>)

**Response**: Thank you for raising a very important and educative point dear! It is obvious that consumption of honey by children under one year has many negative implications related with the toxicity effect of C.botulinum. However, this study mainly focused on the consumption of natural honey by pregnant women and its health and nutrition implications to the mother and her growing fetus before she give birth. Thus, we could not find any evidence which suggests to prevent pregnant mothers from consuming natural honey because of consequences to them and their fetus. The articles you suggested us as well are about consumption of natural honey after delivery and before one year of age.
