# Supplementary material for: Food taboos and related misperceptions during pregnancy in Mekelle city, Tigray, Northern Ethiopia
Source: PLoS One. 2020 Oct 13;15(10):e0239451. doi: 10.1371/journal.pone.0239451 (PMC7553351; doi:10.1371/journal.pone.0239451)
Supplement: Attachment — Submitted filename: Response to reviewers revised version.docx [file pone.0239451.s006.docx]

Response to reviewers

Reviewer #1: As per my previous review of this paper, I believe you report important data with obvious public health and social implications. Further, you have addressed the majority of my specific concerns regarding the need to provide a little more ethnographic context, to unpack some of your measures (e.g. dietary diversity), and the possible benefits of food taboos.

However, the paper still lacks any sort of clear theoretical frame. Maybe this is down to a disciplinary difference? I come from anthropology, and USING AND DEVELOPING THEORY IS ESSENTIAL TO ACADEMIC PUBLICATION. What debate(s) does this study move forward? You claim that you addressed this issue in lines 60-78, but this is just a shallow reading of some evidence suggesting that food taboos may sometimes have benefits, and are variable from society to society. It doesn't offer any suggestion as to WHY taboos vary among populations, and what that might mean for the study population (in terms of their diets, their current health, their future health, the health of the next generation, the wider environmental context in Mekelle). I don't know, man. I would still call this not even close to ready for publication until some deep thought has been put into theory, hypotheses, and predictions. But, on the other hand, I work quite a bit with public health researchers and health scientists and they're not as worried about theory as anthropologists (or sociologists or biologists or psychologists - the home disciplines of my main collaborators) tend to be. So, I guess it'll be up to the editor whether this cuts the mustard?

Many thanks for your constrictive as well as valuable comments. Based on the comments given we tried to clarify why taboos vary from population to population mainly taking into consideration the beliefs and practices of the study area. We also tried to address what would be the possible fate of practicing food taboo to the people in the study area mainly pregnant mothers, its implication to the next generation, and we also hypothesized what would happen if proper intervention is not done. Furthermore, we tried to describe the fate of globalization towards food taboo mainly in the new generation. We have addressed the points in the introduction section of our manuscript in lines; 64-71; 76-100; and 112-116. We also inserted our references for the evidences in the reference list numbers; 7-11 and 14-18.

You seem like you might be interested in food taboos as a possible proximate CAUSE of low dietary diversity during pregnancy. Why don't you test this? Would be a super simple model to run. Do women who report adhering to food taboos have lower dietary diversity scores/ are less likely to meet the MDD cut-off? If so, is this independent of education, control over financial resources? If not, what's the point of the paper? I mean, it'd still be interesting data - you'd just need to figure out a different angle (like, woah, lack of access to control over household resources, for example, is actually a more important driver of low MDD than taboos).

We would like to appreciate your constructive comment. Though it was out of our research objective taking into consideration the importance of your constructive comment, we have analyzed the association between food taboos and MDDS. Based the bivariate analysis, we could observe that the number of pregnant mothers who reported experience of food taboo and had low MDDS were higher than mothers who reported no food taboo and had low MDDS. However, the finding was not statistically significant (31 (11.8%), 7 (10.8%), p value 0.703, respectively. We believe that we could see significant difference among the mothers if our sample size was big. Farther research with bigger sample size to assess the relationship between MDDS and food taboos during pregnancy shall be conducted.

Reviewer #2: 1. Page7, Ln133-134: please, put the numerator and denominator in bracket and multiply it by the “sample size”
2. age9, Ln180: multi-co linearity should be written as “multicolinearity”
3. Page9, Ln181: It is uncommon to use the standard errors to check multicolinearity, VIF is common. Anyway, put a reference for the “standard error of > 2” criterion.
4. Page 11, Ln201 (Table 1): please don’t underline any word in scientific words. Don’t use contractions in research works. So, avoid the contractions like “No” and “≥5 childbirths” and write it like “number” and “≥5 childbirths”. Please, check for such other things throughout the manuscript.

Many thanks for your valuable comments. We have changed the methods of assessing multicolinearity in our model using the commonly used techniques, the variance inflation factor (VIF). Accordingly all independent variables had VIF less that 5 and no variables were excluded from the analyses. We explained this in the data analysis and management section of our manuscript between lines 213-215. We also inserted our reference/evidence for this justification in the reference list number 13. Moreover, we have addressed all the constructive comments given including the grammar (language) checks and other comments in the manuscript.
