# Supplementary material for: Food taboos and related misperceptions during pregnancy in Mekelle city, Tigray, Northern Ethiopia
Source: PLoS One. 2020 Oct 13;15(10):e0239451. doi: 10.1371/journal.pone.0239451 (PMC7553351; doi:10.1371/journal.pone.0239451)
Supplement: Attachment — Submitted filename: Response to reviewers.docx [file pone.0239451.s007.docx]

Response to reviewers

Reviewer #1: Dear Authors,
I think generally you've addressed my most important comments from the previous draft. In particular, you've now framed the background in a way that foregrounds the questions: "why might there be food taboos during pregnancy?" and "How might those taboos affect maternal and child health?". Furthermore, you have run the additional analysis that I suggested you respect to whether diet diversity score is related to reporting following taboos.
I have 2 remaining recommendations though:
1) Actually report the results of the analysis testing for an association between diet quality and food aversions, and then briefly explain in the discussion the lack of relationship (could be insufficient power to detect an effect, could be that socio-economic and/or political factors are much more important drivers of variation in diet quality).

Thank you very much for your valuable and constructive comments

We have incorporated the result of the analysis on the relation between MDDS and practice of food taboos. We put the results on the results section of our manuscript between lines 300 and 302. And on the discussion section from lines 431 up to 439.

2) Get me or someone else who is not part of your team to go through a word doc version of the text and edit/ proofread it. There are too many grammatical, word choice, and phrasing errors to fix by just noting them by line, otherwise I'd do it now with the journal-generated PDF. There are several places where these errors make the argument difficult to parse, so it'd be a much more solid paper with a little editorial TLC.

If you do these 2 things, In my view, it's acceptable/ ready for publication. I don't need to see an additional revision to greenlight it! Well done.

Thank you very much for your valuable comments.

We would love to greatly appreciate for the commitment of our first reviewer Dr.  Luseadra McKerracher for her precious time invested to do the language and scientific edit of our manuscript. Now the manuscript is edited for language and scientific clarity. We would suggest the journal to recognize her commitment in any means if she is interested on it.

Reviewer #2: I congratulate the authors for their extensive review and resubmission! All my previous comments are addressed. However, the authors need to make two minor revisions. The fullstops on Page5, Line 89 and Page5, Line 100 are in "red" color and this is not allowed; change the red colors to black. Please, make sure that there are no such errors throughout your document, including in your supplementary files.

We would like to thank you for your valuable comments.

We have changed the color to black. We also checked and corrected the entire document and all the supplementary tables.
